# The dual role of FSP1 in programmed cell death: resisting ferroptosis in the cell membrane and promoting necroptosis in the nucleus of THP-1 cells

**DOI:** 10.1186/s10020-024-00861-4

**Published:** 2024-07-15

**Authors:** Xiaoqian Tan, Yinling He, Panpan Yu, Yunong Deng, Zhongcheng Xie, Jiami Guo, Qin Hou, Pin Li, Xiaoyan Lin, Siyu Ouyang, Wentao Ma, Yushu Xie, Zilong Guo, Dandan Chen, Zhixia Zhang, Yunyu Zhu, Fei Huang, Ziye Zhao, Cen Zhang, Zhirong Guo, Xi Chen, Tianhong Peng, Liang Li, Wei Xie

**Affiliations:** 1 Department of Physiology, Clinical Anatomy and Reproductive Medicine Application Institute, Hengyang Medical School, University of South China, Hengyang, 421001 Hunan China; 2 School of Basic Medical Sciences, Xiangnan University, Chenzhou, 423000 Hunan China; 3 Class of Clinical Medicine, University of South China, Hengyang, 421001 Hunan China

**Keywords:** FSP1, Ferroptosis, Necroptosis, Myristoylation, THP-1 cells, AML-M5

## Abstract

**Background:**

Acute monocytic leukemia-M5 (AML-M5) remains a challenging disease due to its high morbidity and poor prognosis. In addition to the evidence mentioned earlier, several studies have shown that programmed cell death (PCD) serves a critical function in treatment of AML-M5. However, the role and relationship between ferroptosis and necroptosis in AML-M5 remains unclear.

**Methods:**

THP-1 cells were mainly treated with Erastin and IMP-366. The changes of ferroptosis and necroptosis levels were detected by CCK-8, western blot, quantitative real-time PCR, and electron microscopy. Flow cytometry was applied to detect the ROS and lipid ROS levels. MDA, 4-HNE, GSH and GSSG were assessed by ELISA kits. Intracellular distribution of FSP1 was studied by immunofluorescent staining and western blot.

**Results:**

The addition of the myristoylation inhibitor IMP-366 to erastin-treated acute monocytic leukemia cell line THP-1 cell not only resulted in greater susceptibility to ferroptosis characterized by lipid peroxidation, glutathione (GSH) depletion and mitochondrial shrinkage, as the FSP1 position on membrane was inhibited, but also increased p-RIPK1 and p-MLKL protein expression, as well as a decrease in caspase-8 expression, and triggered the characteristic necroptosis phenomena, including cytoplasmic translucency, mitochondrial swelling, membranous fractures by FSP1 migration into the nucleus via binding importin α2. It is interesting to note that ferroptosis inhibitor fer-1 reversed necroptosis.

**Conclusion:**

We demonstrated that inhibition of myristoylation by IMP-366 is capable of switching ferroptosis and ferroptosis-dependent necroptosis in THP-1 cells. In these findings, FSP1-mediated ferroptosis and necroptosis are described as alternative mechanisms of PCD of THP-1 cells, providing potential therapeutic strategies and targets for AML-M5.

## Introduction

It is well-established that mammalian programmed cell death (PCD), including apoptotic and non-apoptotic forms implicated in various physiological and pathological processes, is regulated by multiple pathways that involve complex molecular and cellular mechanisms (Tang et al. 2019). To a certain extent, we made an effort to summarized molecular and cellular mechanisms of ferroptosis in previously published reviews (Ouyang et al. 2021; Lin et al. 2022; Li et al. 2023). Ferroptosis is a unique non-apoptotic form of PCD characterized by oxidative stress-induced incorporation of polyunsaturated fatty acids into cellular membranes caused by intracellular iron catalytic activity or endogenous lipophilic antioxidant collapse (Dixon et al. 2012). Necroptosis, characterized by necrotic cell morphology, is generally viewed as an uncontrolled process leading to plasma membrane rupture and swelling of organelles (Lin et al. 2016). Current evidence suggests that necroptosis is triggered by the activation of death receptors and is executed by the necrosome, a regulatory complex containing the receptor-interacting protein kinase 1 (RIPK1), receptor-interacting protein kinase 3 (RIPK3) and mixed lineage kinase domain-like protein (MLKL) (Weindel et al. 2022). Ferroptosis and necroptosis have been identified as potential drivers of nerve injury, ischemia–reperfusion injury, and kidney degeneration, suggesting inhibitors of these processes have huge potential as novel drug candidates (Park et al. 2021; Yuan et al. 2022; Tonnus et al. 2021). In contrast, it is widely thought that induction of these two types of cell deaths represents a potent anticancer strategy (Basit et al. 2017). Although ferroptosis and necroptosis represent promising therapeutic agents against acute myeloid leukemia-M5 (AML-M5) (Du et al. 2019; Xin et al. 2017), a unique subtype of acute myeloid leukemia (AML), the specific mechanism warrants further clarification.

Past studies have shown that various pathways associated with the initiation of ferroptosis ultimately converge to suppress cystine import through the inhibition of cystine-glutamate antiporter (system Xc^−^), which is reportedly necessary for glutathione biosynthesis (Dixon et al. 2012; Yang et al. 2014). Among the existing system Xc^−^ inhibitors, erastin, a potent metabolically stable small molecule, has emerged as a therapeutic strategy to trigger cancer cell ferroptosis and has huge prospects for in vivo and in vitro applications (Wang et al. 2020), but sensitivity to erastin varies greatly across cancer cell lines. A study reported that erastin did not induce ferroptosis in acute monocytic leukemia THP-1 cell lines (Yu et al. 2015), suggesting that additional factors govern resistance to ferroptotic THP1 cell death in evolutionarily developed ways. Two important back-to-back studies by Doll et al. and Bersuker et al. documented a novel suppressor of ferroptosis-ferroptosis suppressor protein 1 (FSP1). FSP1, known as a member of the apoptosis-inducing factor (AIF) family, is a key component of a non-mitochondrial coenzyme Q (CoQ) antioxidant system in the cytomembrane reported to counter lethal peroxidation and ferroptosis independently of the canonical glutathione-based system Xc^−^/GPX4 axis (Bersuker et al. 2019; Doll et al. 2019; Yang et al. 2022). Notebaly, our prior study also confirmed that FSP1 is able to anti-ferroptosis independent of SCL7A11/GPX4 axis in VSMCs (You et al. 2023). However, whether FSP1 plays a key role in defending THP-1 cells against death and the underlying mechanisms remain unclear.

**Fig. 1:**
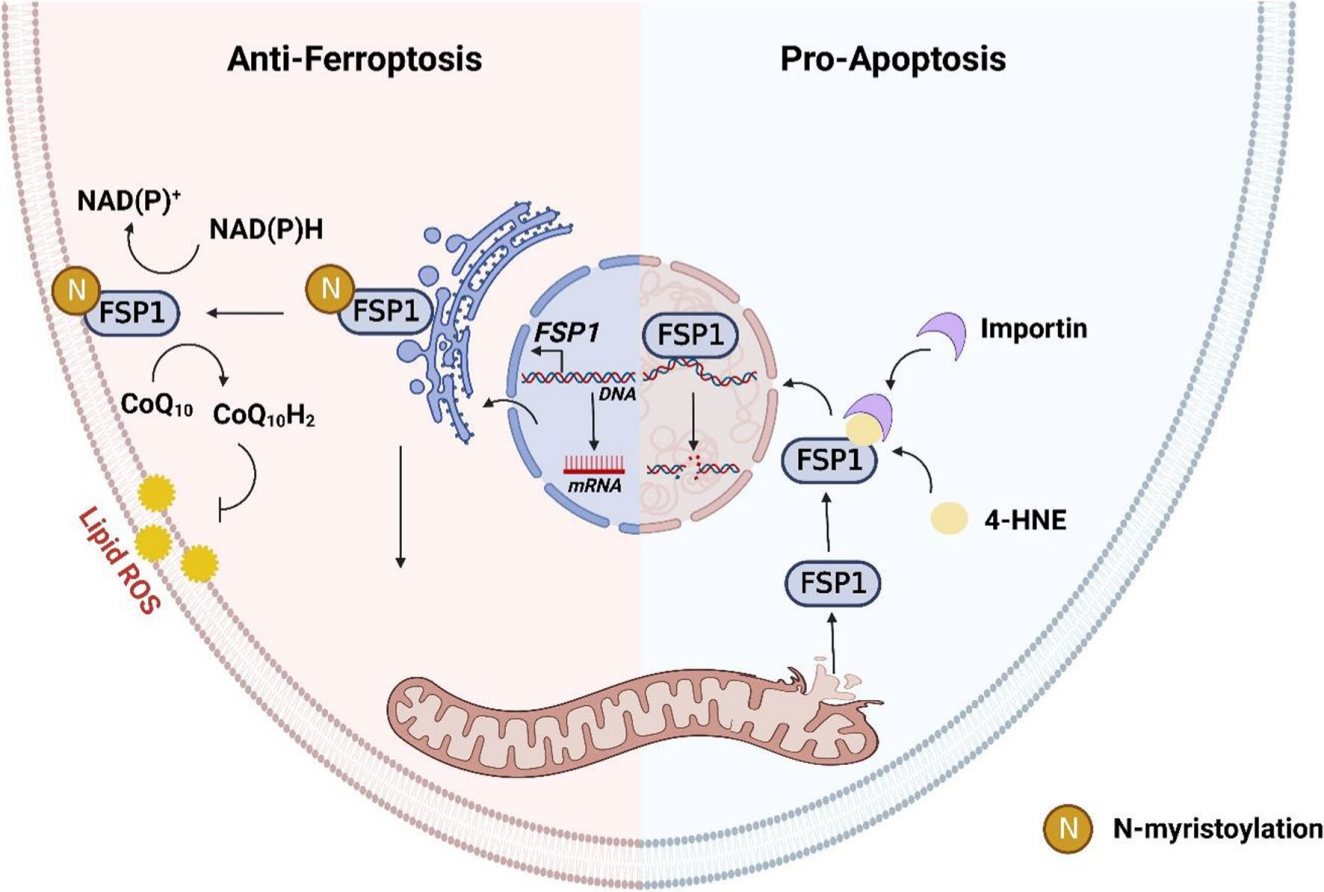
The mechanism of FSP1 in regulating apoptosis and ferroptosis. FSP1 exerts an anti-ferroptosis effect by reducing lipid peroxides through its oxidoreductase activity after anchoring into the cell membrane. However, when FSP1 is transferred from mitochondria to the nucleus during cell apoptosis, it leads to DNA breakage and promotes cell apoptosis via characteristics of non-specific binding to DNA

Protein N-myristoylation is a ubiquitous cotranslational and posttranslational modification catalyzed by myristoyl-CoA: protein *N*-myristoyltransferase (NMT), which attaches myristate, a unique 14-carbon saturated fatty acid, to the N-terminal glycine of various eukaryotic and viral proteins. This protein modification triggers dynamic protein–protein and protein-membrane interactions implicated in diverse physiological processes (Peitzsch and McLaughlin 1993). It is now understood that myristoylation recruits FSP1 to the plasma membrane, where it functions as an oxidoreductase that reduces ubiquinone to ubiquinol, generating a lipophilic radical-trapping antioxidant (RTA) that counteracts lethal lipid peroxidation. Accordingly, myristoylation of FSP1 is essential in protecting against ferroptosis (Doll et al. 2019). Inhibition of NMT by IMP-366 has been shown to induce apoptosis in cancer cell lines by impacting the global myristoylation of lymphoma cell proteins (Kallemeijn et al. 2019; Beauchamp et al. 2020), which has also been documented in endoplasmic reticulum stress (Thinon et al. 2016). Previous studies have demonstrated that the translocation of FSP1 to the nucleus mediated by importin α2 (Imp-α2) could lead to oxidative stress adduction and myocardial apoptosis (Miriyala et al. 2016). Consistently, AIF migration into the nucleus and binding to nuclear DNA have been reported to cause chromosomal condensation and large-scale DNA fragmentation (Daugas et al. 2000; Ye et al. 2002) (Fig. 1). However, whether inhibition of NMT plays a key role in FSP1-mediated regulation of ferroptosis and necroptosis remains unclear. Our study showed that inhibitory myristoylation by IMP-366 could induce THP-1 cell necroptosis by FSP1 migration into the nucleus in a ferroptosis-dependent manner. This highlights the complexity of systems involved in the relationship between ferroptosis and necroptosis, which are responsible for the susceptibility of cells to death under different circumstances.

## Results

### Inhibitory myristoylation by IMP-366 increases susceptibility to erastin-induced ferroptosis in THP-1 cells

**Fig. 2:**
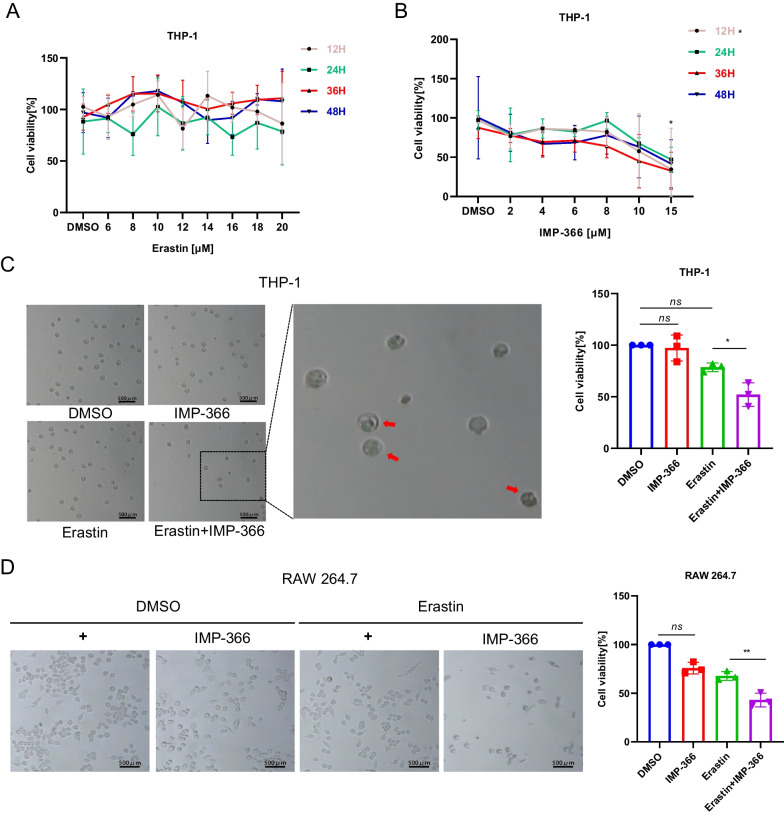

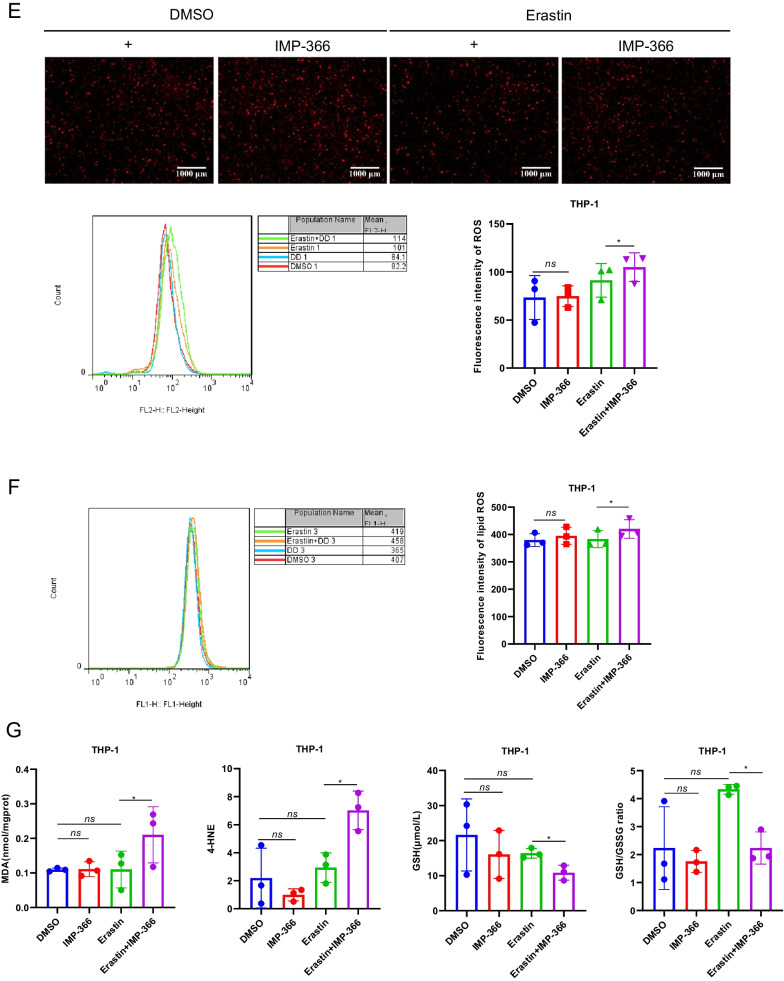

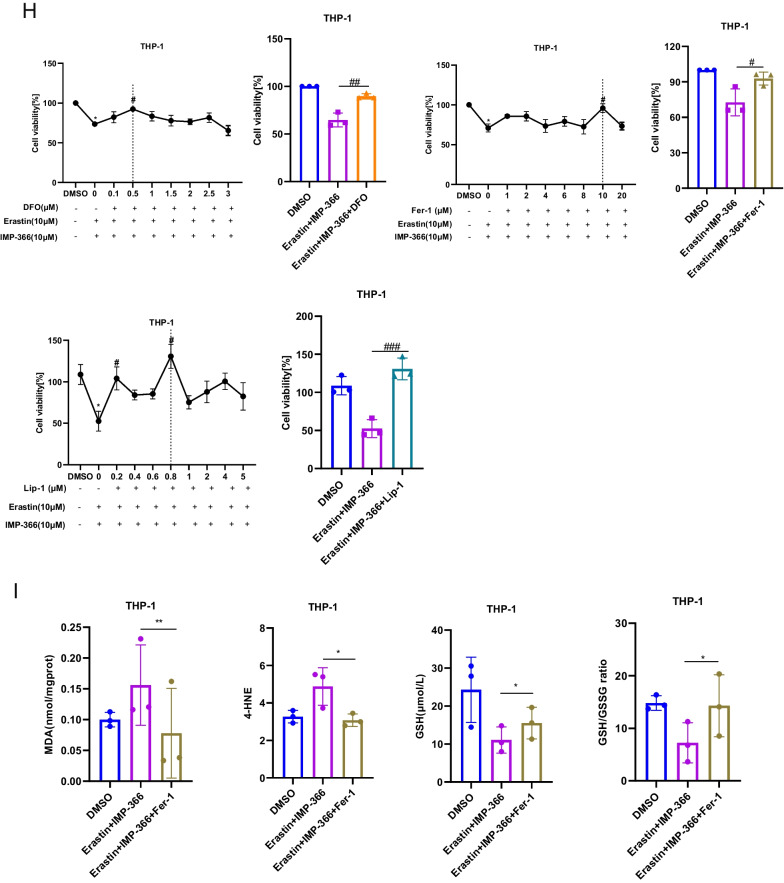

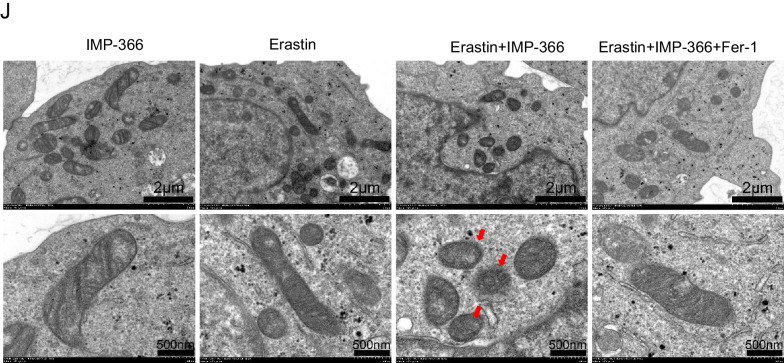
Inhibitory myristoylation by IMP-366 increases susceptibility to erastin-induced ferroptosis in THP-1 cells. **A**, **B** Dose-dependent and time-dependent cytotoxicity of Erastin or IMP-366 in THP-1 cells. Cell viability is expressed as a percentage of the blank control group.*p < 0.05 versus the solvent control group, n = 3. **C**, **D** Cell morphology and cell viability of THP-1 and RAW264.7 cells. After incubating for 36 h with Erastin (10 μM) and/or IMP-366 (10 μM). Cell viability is expressed as a percentage of the solvent control group. *p < 0.05 and **p < 0.01 versus the solvent control group or Erastin-treated group, n = 3. **E**, **F** Flow cytometry with DHE and BODIPY™ 581/591 C11 were separately used to assay ROS level and lipid ROS level in THP-1 cells after administration of 10 μM Erastin and 10 μM IMP-366 for 36 h. Scale bar = 1000 μm, *p < 0.05 versus the solvent control group or Erastin-treated group, n = 3. **G** The MDA and 4-HNE levels were increased, while GSH and GSH/GSSG levels were reduced obviously by Erasitn + IMP-366 treatment for 36 h. *p < 0.05, compared with the solvent control group or Erastin-treated group, n = 3. **H** The cell viability of THP-1 cells was restored significantly by ferroptosis inhibitors including 0.5 μM DFO, 10 μM Fer-1 and 0.8 μM Lip-1. *p < 0.05, compared with the solvent control group. ^#^*p* < 0.05, ^##^p < 0.01 and ^###^*p* < 0.001 ompared with the Erastin + IMP-366-treated group, n = 3. **I** Fer-1 (10 μM) inhibited production of MDA and 4-HNE, and consumption of GSH. *p < 0.05, **p < 0.01 compared with Erastin + IMP-366-treated group at 36 h, n = 3. **J** Ferroptotic ultrastructral changes in THP-1 cells, as acquired by electron microscopy. Red arrows indicate mitochondrial shrinkage and cristae decreased, which could be alleviated by Fer-1, bar: 2 μm and 500 nm. The values shown as represent the means ± SD

Although system Xc^−^ has been identified as an anti-ferroptosis factor, whose activity determines ferroptosis sensitivity (Dixon et al. 2012; Imoto et al. 2018), the inhibitory effect of erastin on system Xc^−^ failed to trigger ferroptosis in THP-1 cells lines (Fig. 2A), suggesting the existence of alternative resistance mechanisms. In THP-1 cell lines treated with a non-lethal dose of IMP-366, an inhibitor of N-myristoyltransferase (Fig. 2B), both qualitative analysis of cell morphology and quantification of cell viability revealed a significant increase in the sensitivity of blocked myristoylation to erastin-induced death (Fig. 2C, D), indicating inhibition of myristoylation in THP-1 cells increased sensitivity to additional ferroptotic inducer erastin, although it remains unclear whether ferroptosis was involved. The role of oxidative stress-induced lipid peroxidation in different types of programmed cell death, such as apoptosis (Valko et al. 2006; Hanikoglu et al. 2020), necroptosis (Basit et al. 2017), and ferroptosis (Stockwell et al. 2017), is well established. This process is driven by excess reactive oxygen species (ROS) that leads to lipid peroxidation chain reactions and damages biomembranes (Oliveira et al. 2019). In the present study, supplementing IMP-366 to cells treated with erastin resulted in increased cytosolic and lipid ROS (Fig. 2E, F) accompanied by upregulation of lipid degradation products malonaldehyde (MDA) and 4-hydroxynonenal (4-HNE), caused by GSH depletion (Fig. 2G). To increase the robustness of these findings, ferroptosis inhibitors were co-administered. The viability of erastin-treated cells whose myristoylation had been inhibited by IMP-366, was rescued by deferoxamine (DFO), ferrostatin-1 (Fer-1), and liproxstatin-1 (Lip-1) (Fig. 2H). Besides, Fer-1 could downregulate the levels of MDA and 4-HNE and inhibit depletion of GSH induced by IMP-366 (Fig. 2I). Electron microscopy images of erastin-treated cells supplemented with IMP-366 exhibited distinct ferroptotic mitochondrial morphology accompanied by shrunken mitochondria, increased membrane density, and reduced mitochondrial cristae, which could be relieved after treatment with Fer-1 (Fig. 2J). These findings demonstrate that IMP-366 increases susceptibility to erastin-induced ferroptosis.

### Inhibition of FSP1 myristoylation increases susceptibility to erastin-induced ferroptosis

**Fig. 3:**
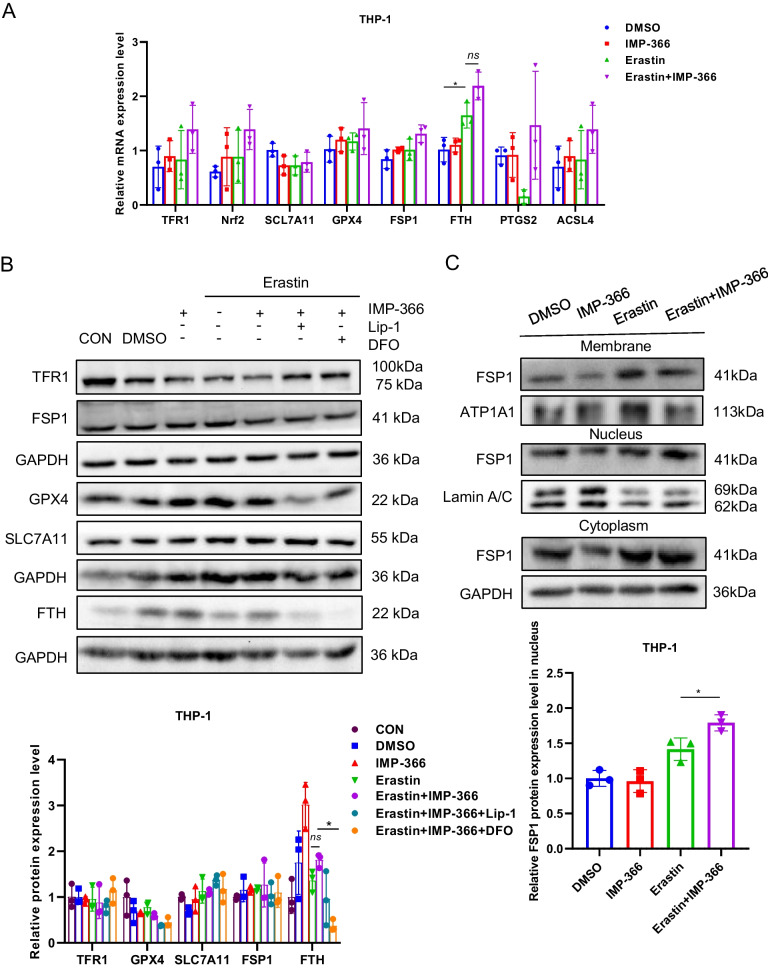

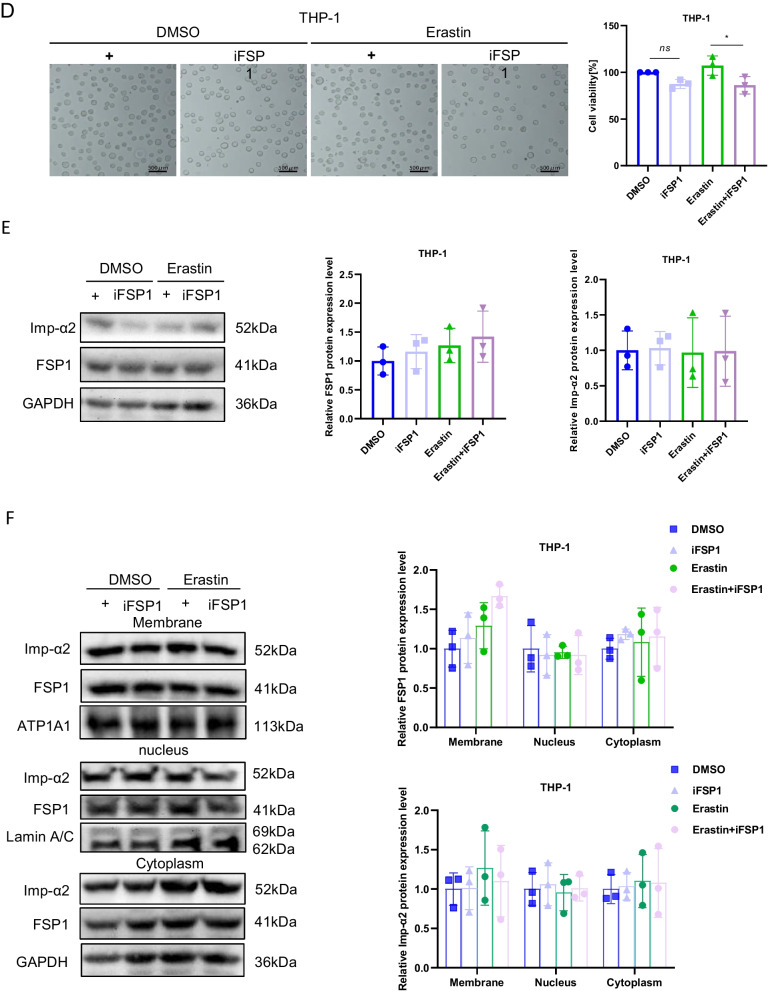

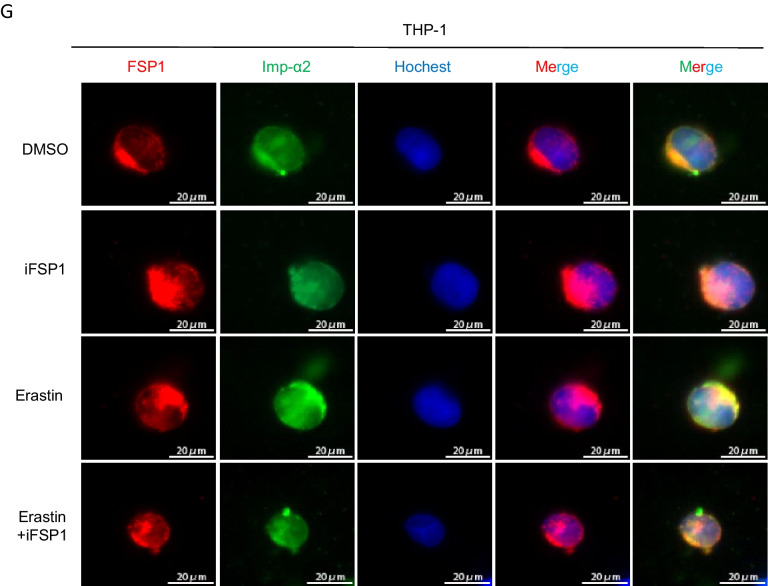
Inhibition of FSP1 myristoylation sensitizes to erastin-induced ferroptosis. The ferroptosis-related factors’ (**A**) mRNA and (**B**) protein expression levels. *p < 0.05 compared with the DMSO control or the Erastin-treated group, n = 3. **C** The FSP1 protein expression levels in whole membrane, nucleus, and cytoplasm of THP-1 cells. Nuclear FSP1 protein levels visibly increased in Erastin + IMP-366-treated group compared with Erastin-treated group, while membranous FSP1 protein levels decreased. **D** Cell morphology and cell viability of THP-1 cells after adding FSP1 inhibitor iFSP1 (3 μM) for 36 h. Scale bar = 500 μm. Cell viability were obviously decreased in Erastin + iFSP1-treated group after inhibiting cell membrane anchoring of FSP1 by iFSP1, compared with Erastin-treated group. *p < 0.05, n = 3, results are shown as means ± SD. **E** iFSP1 hardly affected the expression of FSP1 and (**F**) the distribution of FSP1. All that were measured via western-blotting in THP-1 cells. **G** Determination of localization of FSP1 (red) and Imp-α2 (green) in THP-1 cells via immunofluorescence, nucleus (blue) were detected with Hochest 3344 (scale bar = 20 μm)

It is well-established that the accumulation of lipid peroxides can be driven by severe dysregulation of antioxidant systems (Lei et al. 2020; Wu et al. 2021) and/or iron metabolism disorder manifesting as enhanced iron uptake and impaired iron storage (Cheng et al. 2020; Park and Chung 2019). Interestingly, the mRNA and protein expressions in intracellular transferrin receptor 1 (TFR1), ferritin heavy chain (FTH), or endogenous lipophilic antioxidant systems, such as solute carrier family 7 member 11 (SLC7A11), glutathione peroxidase 4 (GPX4), and FSP1, were not related to IMP-366’s increased susceptibility to ferroptosis (Fig. 3A, B). Interestingly, similar to previous reports that myristoylation recruits FSP1 to the plasmalemma where it functions as an oxidoreductase is necessary to confer ferroptosis resistance (Bersuker et al. 2019; Doll et al. 2019), IMP-366 significantly promoted the transfer of FSP1 from the membrane to the nucleus (Fig. 3C), a phenomenon that was significantly reversed by inhibitors of ferroptosis (Fig. 6C), suggesting sensitization of IMP-366 to erastin-induced ferroptosis is primarily mediated by interference with FSP1 recruitment to the plasmolemma. Inhibition of the oxidoreductase activity of FSP1 with iFSP1 increased the sensitivity of THP1 cells to erastin-induced death (Fig. 3D) without affecting the expression (Fig. 3E) and importin α2-mediated translocation of FSP1 into the nucleus (Fig. 3F, G), indicating deficiency of the FSP1 oxidoreductase activity on the plasmalemma is vital for sensitization to erastin-induced ferroptosis.

### The pro-necroptosis role of IMP-366-mediated translocation of FSP1 to the nucleus

**Fig. 4:**
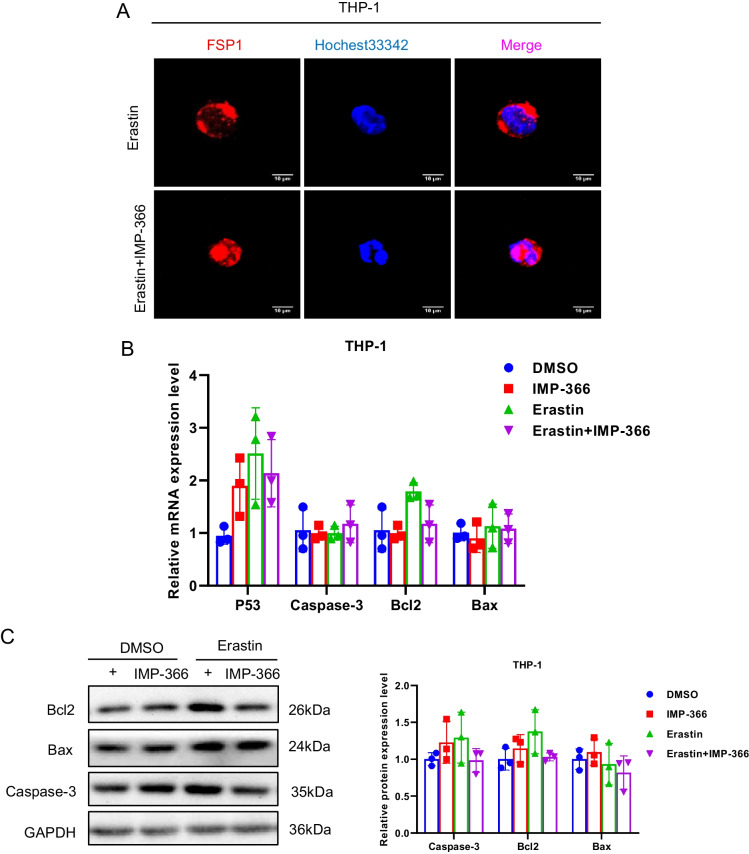

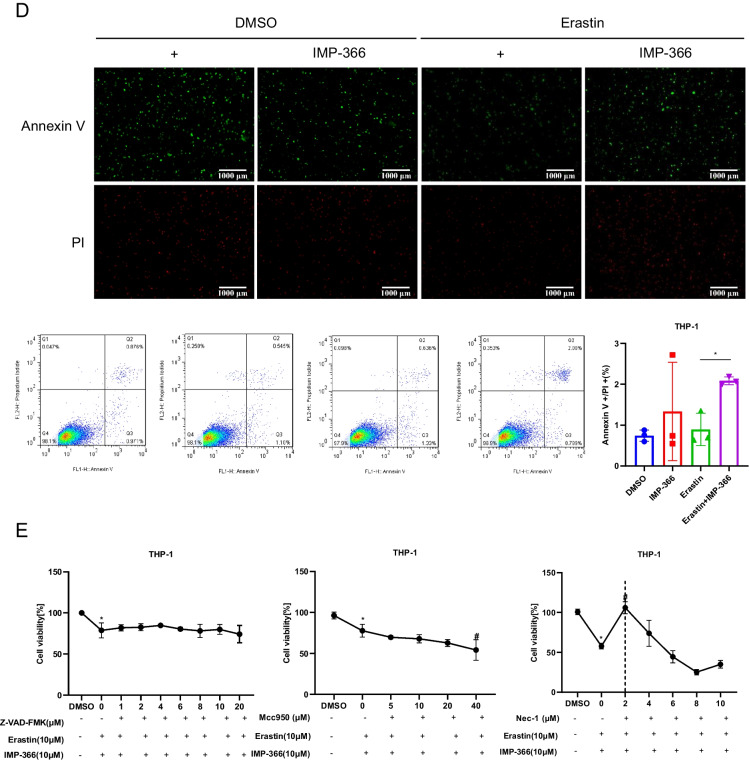

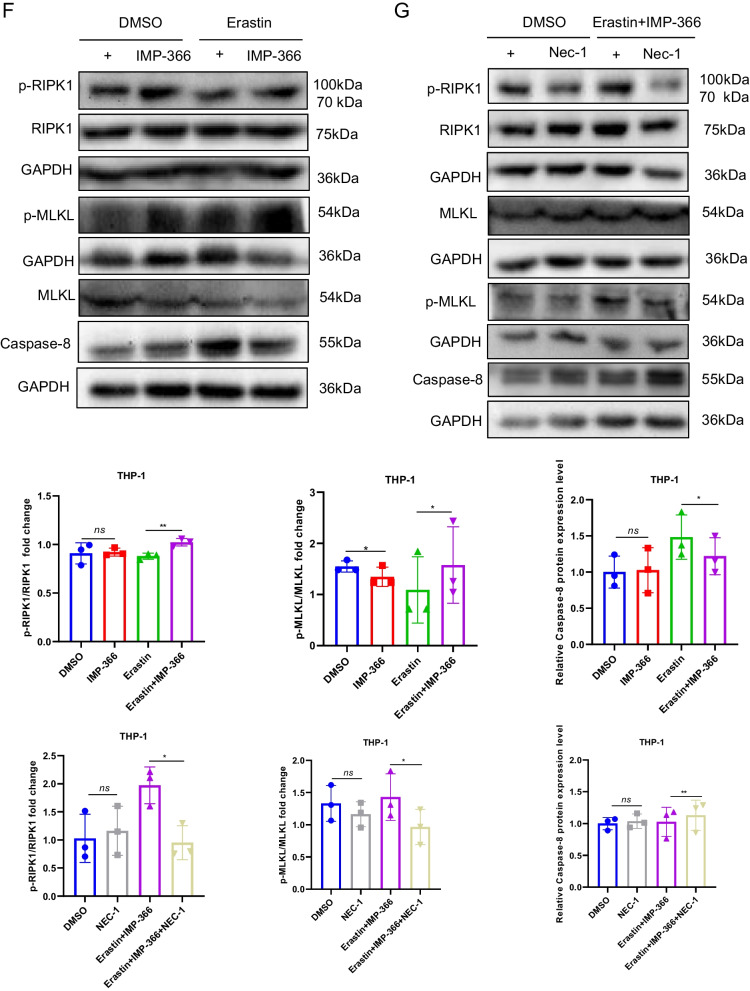

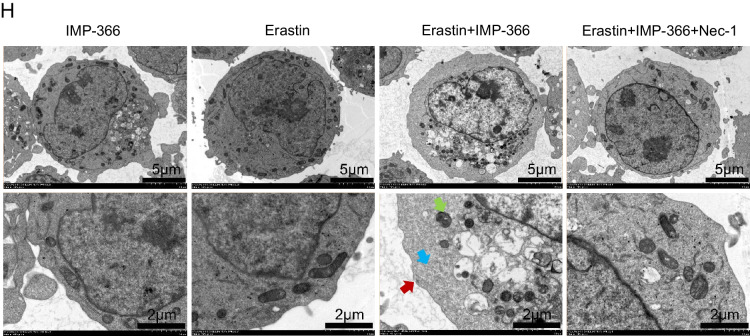
The pro-necroptosis role of IMP-366-mediated FSP1 nuclear translocation. **A** The subcellular localization of FSP1 (red) and the morphology of the nucleus (blue) after treatment with Erastin and IMP-366 in THP-1 cells were detected via laser scanning confocal microscopy confocal microscope. All the bars shown 10 µm. **B** The mRNA levels and (**C**) western blot analysis of apoptosis-related factors shown no difference between the Erastin + IMP-366-treated group and the Erastin-treated group, n = 3. **D** Representative images of PI (red) and annexin V (green), and flow cytometry images of THP-1 cells stained with PI/annexinV, scale bar: 1000 μm. The ratio of PI + /annexinV + represented necroptosis activation, *p < 0.05 versus the Erastin-treated group, n = 3. **E** The cell viability of THP-1 cells treated with Erastin + IMP-366 could be rescued by necroptosis inhibitor nec-1 (2 μm), while apoptosis inhibitor z-VAD-fmk and pyroptosis inhibitor mcc950 failed. Data were detected via CCK-8 assay, *p < 0.05, compared with the solvent control group. ^#^p < 0.05, compared with the the Erastin + IMP-366-treated group. n = 3. **F**, **G** Expression levels of p-RIPK1/RIPK1, p-MLKL/MLKL and caspase-8 in THP-1 cells. *p < 0.05 versus the Erastin-treated group or the Erastin + IMP-366-treated group, n = 3. **H** Representative electron microscopy images were conducted to determine typical morphological changes of necroptosis in THP-1 cells. Red arrow: membrane breakdown, Blue arrow: translucent cytoplasm, Green arrow: swelling mitochondria. The bars indicated 2 µm and 5 µm

Consistent with previous reports that induction of cell apoptosis independent of caspase following NMT inhibition (Beauchamp et al. 2020; Thinon et al. 2016) may be closely related to FSP1 accumulation in the nucleus (Bilyy et al. 2008; Tan et al. 2020). IMP-366 was found to induce FSP1 nuclear translocation and non-specific combination with DNA accompanied by chromatin condensation (Figs. 3C, 4A) without changes in the expression of apoptosis-related factors p53, caspase-3, bcl2 and bax (Fig. 4B, C). In addition, annexin V/PI flow cytometry results showed IMP-366 significantly increased the number of cells stained with PI/annexin V (Fig. 4D). However, cell death was not suppressed by the apoptosis inhibitor Z-VAD-FMK and pyroptosis inhibitor MCC950, unlike the necroptosis inhibitor necrostatin-1 (Nec-1) (Fig. 4E). The ratio of p-RIPK1/RIPK1 and p-MLKL/MLKL was significantly increased, while the expression of caspase-8 was reduced (Fig. 4F), suggesting that necroptosis activation (Oberst et al. 2011) could be reversed by Nec-1 (Fig. 4G) in erastin-treated cells supplemented with IMP-366. According to the electron microscopy images of the periinfarct area, the characteristic cytological changes of necroptosis were induced by IMP-366 including translucent cytoplasm, swelling mitochondria, and membrane breakdown, (Chen et al. 2019). However, the administration of Nec-1 could alleviate these changes, indicating that the impact of IMP-366 on THP-1 cells may be attributed to necroptosis rather than apoptosis or pyroptosis (Fig. 4H). These findings substantiate that IMP-366 increases cellular susceptibility to erastin-induced ferroptosis and necroptosis in THP-1 cells, although the exact mechanism remains elusive.

### IMP-366 promotes necroptosis by recruitment and transfer of FSP1 into the nucleus by importin α2

**Fig. 5:**
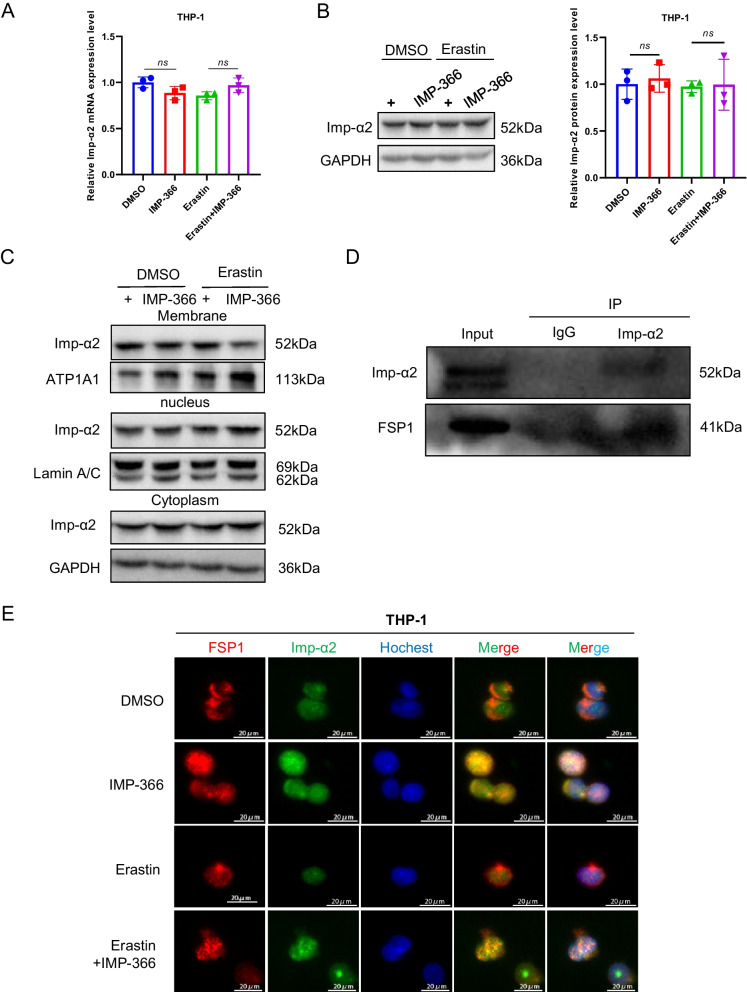

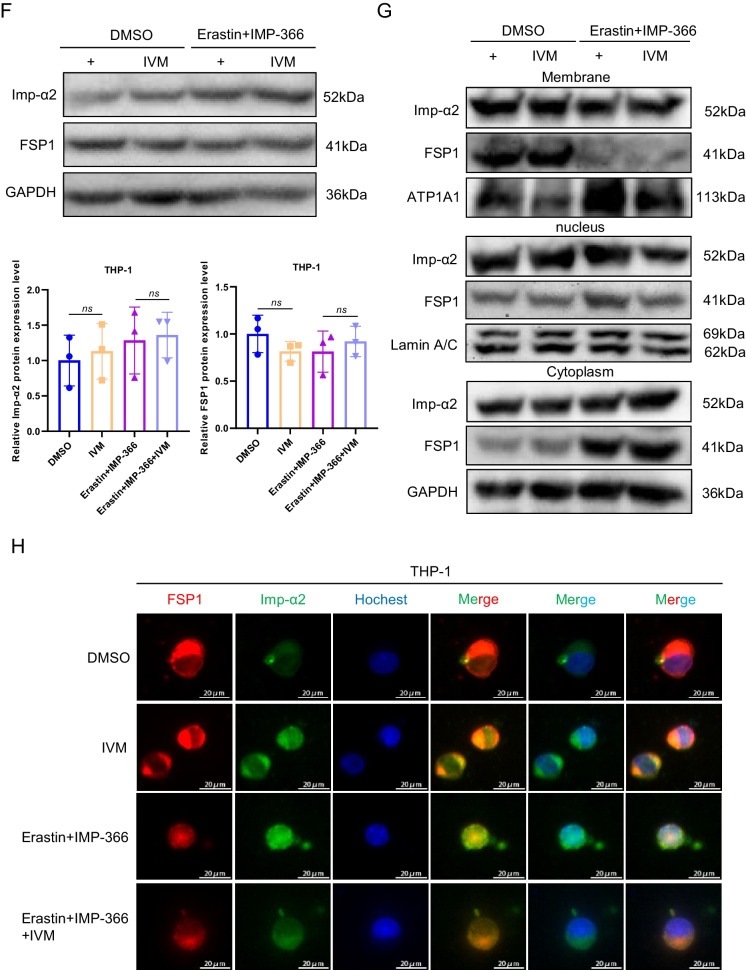

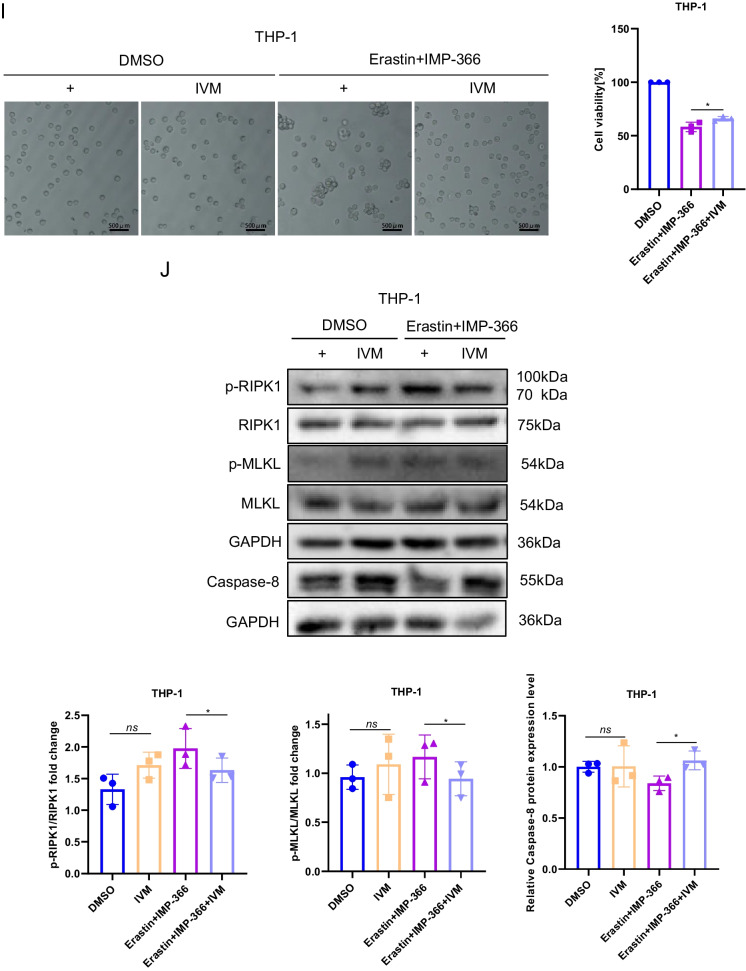

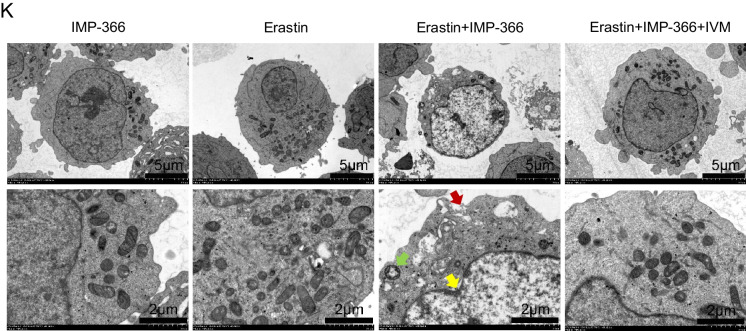
IMP-366 promotes necroptosis by recruitment and transfer of FSP1 into the nucleus by importin α2. **A** The mRNA expression of Imp-α2 detected via qPCR shown no significant difference, and (**B**) the protein expression levels measured with relative gray values were also consistent. NS, no significant difference versus the DMSO-treated group or the Erastin-treated group, n = 3. **C** The effect of Erastin + IMP-366 treatment upregulation on nucleus Imp-α2 distribution and downregulation on membranous Imp-α2 distribution, compared with the effect of the Erastin-treated group in THP-1 cells. **D** Immunoprecipitation analysis was performed to detect the potential interaction between FSP1 and Imp-α2 in THP-1 cells. **E** FSP1 fluorescence (red) and Imp-α2 fluorescence (green) were used to indicate protiens distribution and co-location in different treatment of THP-1 cells. Nucleus (blue) were detected by hochest 3344. Bar = 20 μm. **F** The effect of IVM (15 nm) on FSP1 and Imp-α2 protein expression. ns, no significant difference vs. the DMSO-treated group or the Erastin + IMP-366 group, n = 3. **G** The effect of IVM (15 nm) on distribution of FSP1 and Imp-α2 protein in THP-1 cells. **H** Immunofluorescence shown that the addition of IVM (15 nm) prevented Imp-α2 (green) from transferring FSP1 (red) into the nucleus, compared with Erastin + IMP-366-treated group. Bar = 20 μm. **I** Left: Microscopic images of cell morphology in different groups (Bar = 500 μm). Right: cell viability of THP-1 cells treated with Erastin + IMP-366 were rescued by 15 nm IVM. *p < 0.05, compared with the Erastin + IMP-366-treated group, n = 3. **J** Representative Western bands showing that IVM (15 nm) downregulated the expression level of p-RIPK1/RIPK1, p-MLKL/MLKL, and upregulated the protein expression of caspase-8. *NS* no significant difference compared with the DMSO group, *p < 0.05, compared with the Erastin + IMP-366 group, n = 3. Results are shown as means ± SD. **K** Representative necrotic morphology images of THP-1 cells by transmission electron microscopy (TEM). Red arrow indicates membrane breakdown, green arrow indicates swelling mitochondria, and yellow arrow indicates perinuclear space widening. Scale bar: 2 μm and 5 μm

To investigate the mechanism underlying the translocation of FSP1 to the nucleus and its role in cell death (Miriyala et al. 2016), we conducted expression analysis and localization studies of importin proteins due to their capacity to recognize FSP1 and facilitate its transportation into the nucleus. Importin α2 exhibited an upregulation in nuclear localization without a significant change in overall expression following IMP-366 treatment (Fig. 5A–C). Consistently, FSP1 exhibited significant nucleus co-localization via binding with importin α2 (Fig. 5D) in experimental groups treated with IMP-366 (Fig. 5E). These results collectively revealed that importin α2 is essential for FSP1 nucleus transposition.

To confirm whether importin α2-mediated FSP1 nucleus translocation induced necroptosis, the interaction of FSP1 and importin α2 was suppressed by ivermectin (IVM), a potential inhibitor of importin α/β-mediated transport (Miriyala et al. 2016; Yang et al. 2020). IVM could reverse the IMP-366-induced increase in importin α2 and FSP1 in the nucleus without an increase in importin α2 and FSP1 expression (Fig. 5F–H). Furthermore, the decrease in cell viability caused by this increase was reversed by IVM (Fig. 5I). In addition, the ratio of p-RIPK1/RIPK1 and p-MLKL/MLKL decreased, and caspase-8 was increased by IVM treatment (Fig. 5J), consistent with electron microscopy findings that ivermectin suppressed necroptosis (Fig. 5K). These results indicated that FSP1 nucleus translocation via importin α2 is an inevitable event of necroptosis facilitated by IMP-366 in erastin-treated cells.

### IMP-366 induces necroptosis by FSP1 migration into the nucleus in a ferroptosis-dependent manner

**Fig. 6:**
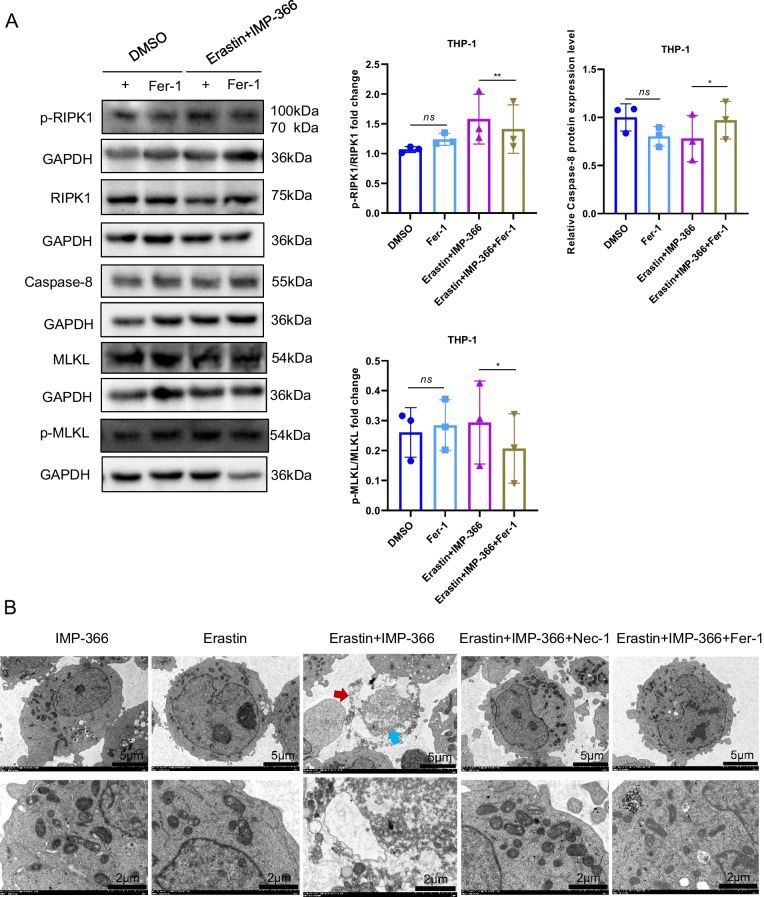

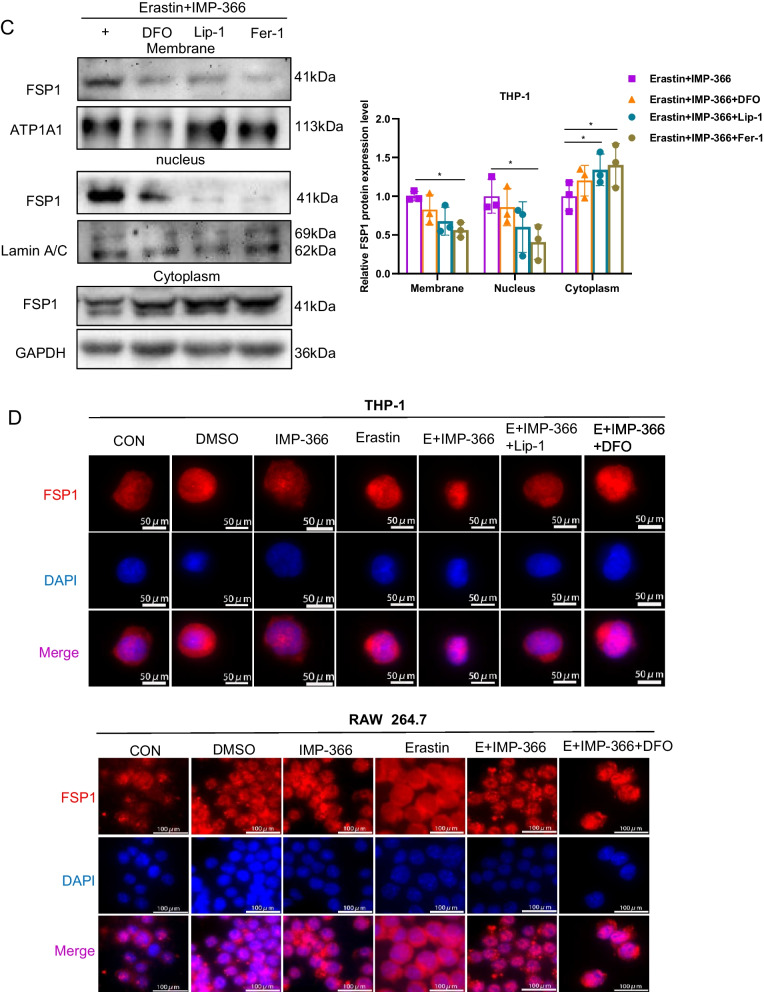

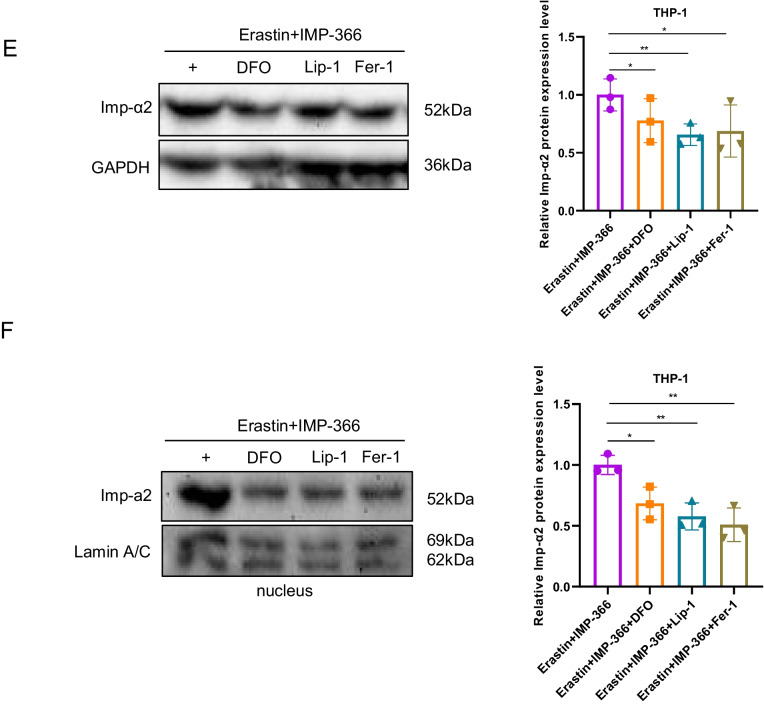
IMP-366 induces necroptosis by FSP1 migration into the nucleus in a ferroptosis-dependent manner. **A** Representative western blotting bands and quantitative analyses of necroptosis markers after adding ferroptosis inhibitor Fer-1. NS, no significant difference compared with the DMSO group, *p < 0.05, compared with the Erastin + IMP-366 group, n = 3. **B** Representative necroptosis cell morphology exhibiting membrane breakdown (red arrow) and nucleus fragmentation (blue arrow) were both rescued by ferroptosis inhibitor Fer-1, similar to necroptosis inhibitor Nec-1. The effect of ferroptosis inhibitors on distribution of FSP1 indicated by (**C**) western blotting bands and (**D**) immunofluorescence staining of FSP1 (red), nucleus (blue) were stained with DAPI. *p < 0.05, compared with the Erastin + IMP-366-treated group, n = 3. **E**, **F** The effect of ferroptosis inhibitors on exreprssion and nucleus distribution of Imp-α2 protein. *p < 0.05, **p < 0.01, compared with the Erastin + IMP-366-treated group, n = 3. Results are shown as means ± SD

We established that IMP-366 facilitates FSP1 migration from the membrane to the nucleus and induces necroptosis in erastin-treated cells. Interestingly, ferroptosis inhibitors could salvage cell activity (Fig. 2H), suggesting a potential relationship between ferroptosis and necroptosis. These results validated that Fer-1, a ferroptotic inhibitor, could reverse IMP-366-induced upregulation of necroptotic markers and the morphological changes associated with necroptosis (Fig. 6A, B), indicating IMP-366-mediated necroptosis is dependent on ferroptosis. In addition, IMP-366-induced FSP1 nucleus localization could be reversed by ferroptotic inhibitors (Fig. 6C, D) accompanied by reduced overall and nuclear content of importin α2 (Fig. 6E, F). These results provide compelling evidence that IMP-366 could accelerate necroptosis in a ferroptosis-dependent manner based on importin α2-mediated FSP1 migration to the nucleus.

## Discussion

**Fig. 7:**
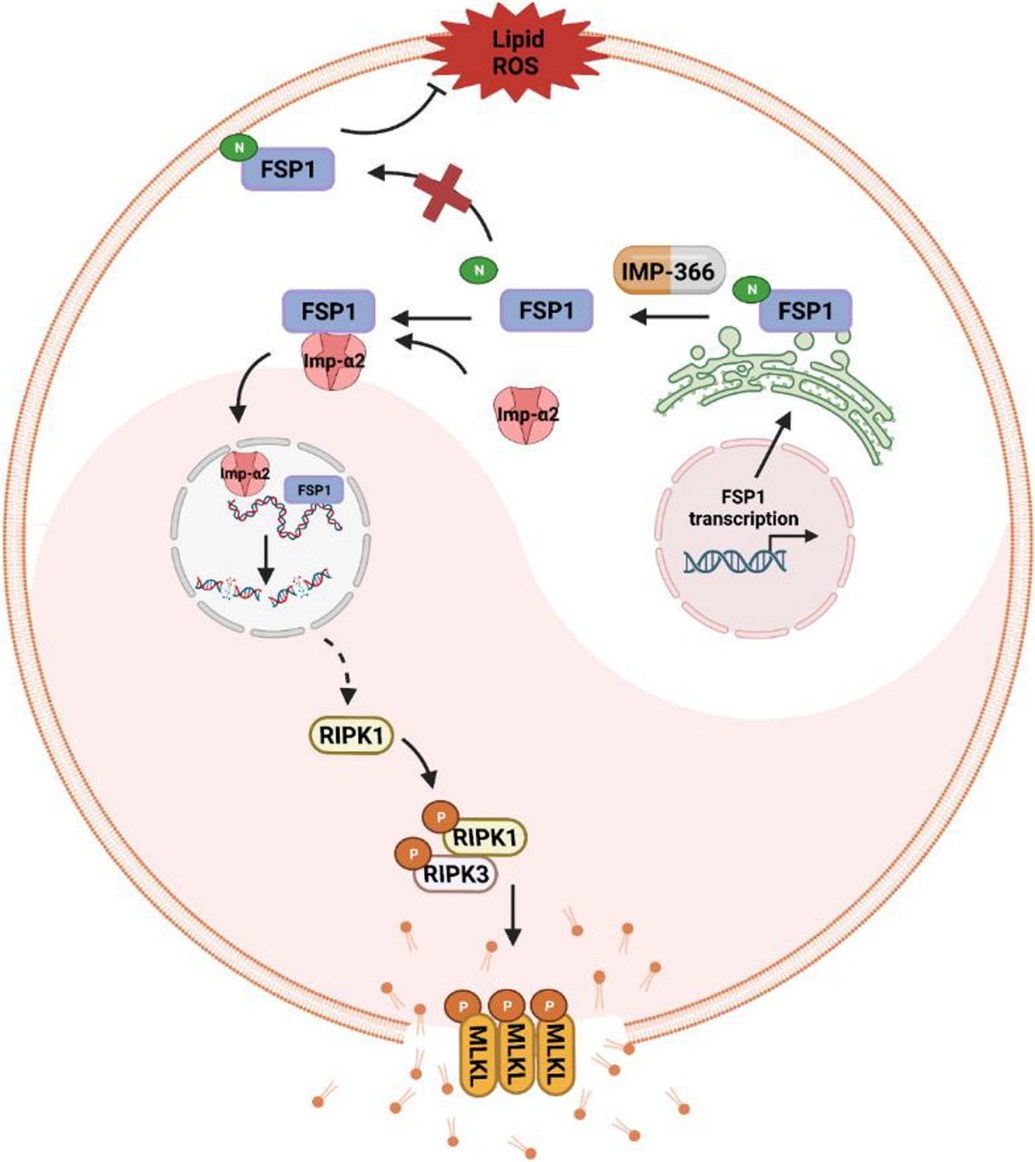
Graphical abstract depicting the anti-ferroptosis and pro-necroptotic functions of FSP1. The anti-ferroptosis function of FSP1 is dependent on *N*-myristoylation, which is essential for FSP1 to target the plasma membrane, where FSP1 prevents lipid peroxidation. Myristoylation inhibitor IMP-366 abolishes the anti-ferroptosis function of FSP1 and contributes to the nuclear transposition of FSP1 via importin α2. Excessive FSP1 migrates into the nucleus, activating cell necroptosis

While ferroptosis and necroptosis are distinct pathways of genetically regulated programmed cell death, many previous experimental studies have revealed common ferroptotic and necroptotic cell death characteristics in the same cellular models or lineages (Basit et al. 2017; Chu et al. 2022; Zille et al. 2017; Muller et al. 2017), which were also observed in the present study. However, the relationship between ferroptosis and necroptosis remains controversial, and the mediator that triggers both pathways remains unknown. Herein, we substantiated that necroptosis and ferroptosis are induced instead of apoptosis and pyroptosis. Our findings also suggested the induction of necroptosis during ferroptosis in erastin-treated acute monocytic leukemia THP-1 cell lines after treatment with an inhibitor of N-myristoyltransferase IMP-366. Based on these findings, it is highly conceivable that IMP-366 effectively triggers necroptotic signaling, reaching the required threshold for cellular death. Moreover, in the existence of IMP-366these pathways may lead to ferroptosis, owing to the insufficient presence of FSP1 in the cell membrane (Fig. 7).

The dependence of necroptosis on ferroptosis has attracted significant attention, primarily directed towards the central regulator FSP1, which mitigates ferroptosis by means of its antioxidant capacity to hinder lipid peroxidation. FSP1 engages not only in cellular differentiation and glycolipid metabolism but also modulates programmed cell death, thereby facilitating accurate regulation of physiological and pathological processes of the organism (Nguyen et al. 2020; Gong et al. 2007). The heterogeneous distribution and localization of intracellular FSP1 contribute to its different functions. FSP1 in the membrane of mitochondria acts as a mitochondrion-associated NAD(P)H oxidase, contributes glycolysis by supplementing hydrogen acceptor NAD^+^, and supports thermogenesis by providing electrons (Nguyen et al. 2020). Moreover, there is an increasing consensus that FSP1 significantly reduces ROS and promotes the differentiation of oocyte cells and brown adipocytes, although the specific molecular mechanism remains unclear (Nguyen et al. 2020; Shao 2015). Interestingly, FSP1 has been redefined as a vital factor dependent on N-myristoylation, which enables FSP1 to anchor in the membrane where FSP1 plays an anti-ferroptosis role by counteracting lethal phospholipid peroxidation dependent on its oxidoreductase activity (Bersuker et al. 2019; Doll et al. 2019). In the present study, we validated that suppressing N-myristoylation of FSP1 by IMP-366 could abolish anti-ferroptosis in erastin-treated THP-1 cells. In addition, once translocated into the nucleus, FSP1 could trigger caspase-independent apoptosis through non-specific binding to DNA, thereby leading to DNA fragmentation (Yang et al. 2011; Marshall et al. 2005; Wu et al. 2002). Consistent with the literature, our findings corroborated increased FSP1 distribution in the nucleus and significantly reduced cell viability in THP-1 cells treated with myristoylation inhibitors. Intriguingly, THP-1 cells exhibited necroptosis but not apoptosis, this phenomenon could be rescued by a ferroptosis inhibitor, indicating necroptosis-induced FSP1 migration into the nucleus in a ferroptosis-dependent manner. Overall, our findings suggest that ferroptosis triggers the initiation of necroptotic factor transcription mediated by the abundance of FSP1 in the nucleus. Additionally, necroptotic signaling pathways could be activated by a complex formed by FSP1 and importin α2.

The contrasting regulation of FSP1 in ferroptosis and necroptosis, modulated by its differential intracellular localization, could be linked to factors such as its oxidoreductase activity or the distinct distribution of enzymes and coenzymes within cytoplasmic or organelle compartments. Precise regulation of differential distribution or expression of cytokines or signaling in intracellular and even subcellular structures may play a critical role in regulating FSP1-mediated programmed cell death. Given its unique dual role as an anti-ferroptosis and pro-necroptosis agent, regulating the expression and subcellular localization of FSP1 in different pathological states represents a key step toward developing effective prognostic and treatment strategies for related diseases. Indeed, in the realm of cancer therapy, targeting the FSP1-mediated anti-ferroptosis effect and promoting its pro-necroptosis or pro-apoptosis activity could enhance the sensitivity of tumor cells to radiotherapy and chemotherapy, thus providing a promising approach to overcoming drug and radiotherapy resistance.

## Limitation

According to our findings, anti-ferroptosis role of FSP1 is interrupted by inhibiting cell membrane location of FSP1 with the aid of myristoylation inhibitor IMP-366, and then increased nuclear transposition of FSP1 led to ferroptosis-dependent necroptosis in Erastin-cultured leukemic THP-1 cells. Although the dual role of FSP1 in cell death pathways suggests that FSP1 exhibits great potential for AML-M5 therapy, whether similar effects of FSP1 exist in other forms of leukemia or solid tumors still need to be explored. Besides, it is important to determine potential resistance mechanisms after inhibiting FSP1 for a long time and to identify deeper mechanisms associated with necroptosis medicated by FSP1 in more clinical cancer models.

## Materials and methods

### Cell culture

Human acute monocytic leukemia cells, THP1, and the mouse leukemic monocyte cell line, RAW264.7, were cultured using RPMI-1640 medium modified to exclude calcium nitrate and supplemented with 2.05 mM L-Glutamine (Gibco, C11875500BT), 10% fetal bovine serum (FBS) (ExCell, FSP500), and a 1% penicillin–streptomycin mixed solution (Abiowell, AWH0529a). Cells were treated with Erastin (Selleck, S7242), IMP-366 (AOBIOUS, AOB6657), DFO (Sigma, D9533), Fer-1 (Selleck, S7243), Lip-1 (Selleck, S7699), iFSP1 (Cayman Chemical, 29483), Z-VAD-FMK (Selleck, S7023), MCC950 (Selleck, S8930), Nec-1 (Selleck, S8037), and IVM (APExBIO, A2813).

### Western blotting

Cells were lysed in RIPA buffer (Solarbio, R0020) containing 1% PMSF (Solarbio, P0100). Protein fractions (cytoplasm, nucleus, and membrane) were extracted using a protein extraction kit (KeyGEN BioTECH, KGBSP002). Protein samples (25–50 µg) were separated using 10% SDS-PAGE and transferred to polyvinylidene fluoride (PVDF) membranes. The membranes were blocked with 5% low-fat milk in PBST for 120 min at room temperature. Next, the membranes were incubated with primary antibodies overnight at 4 °C, followed by HRP-labeled mouse or rabbit secondary antibodies for 120 min at 4 °C. Labeled proteins were visualized using an automatic chemiluminescence image analysis system (Tanon-5500) after treatment with developer (BOSTER, AR1173). ImageJ was used for quantification. Antibodies and their respective dilutions were as follows: FSP1 (1:2000, Proteintech, 20886-1-AP); TFR1 (1:1000, ABclonal, A5865); GPX4 (1:2000, Abcam, ab125066); SLC7A11 (1:2000, Abcam, ab175186); FTH (1:1000, BOSTER Biological Technology, BM4487); Importin α2 (1:1000, Santa Cruz, SC-55538); Bcl2 (1:1000, PTMab, PTM-5202); Bax (1:1000, Abmart, T40051); Caspase-3 (1:1000, Abmart, T40044F); RIPK1 (1:1000, Abmart, TA7877); Phospho-RIPK1 (Ser161) (1:1000, PTMab, PTM-6624); MLKL (1:1000, Abmart, TP73002F; ABconal, A5579); Phospho-MLKL (1:1000, Abmart, T57146S; ABconal, AP0949); Caspase-8 (1:2000, PTMab, PTM-6085); ATP1A1 (1:1000, Proteintech, 55187-1-AP); Lamin A/C (1:1000, Proteintech, 10298-1-AP); GAPDH (1:5000, ABclonal, AC002); Anti-Mouse IgG (H + L) (1:5000, ABconal, AS003); Anti-Rabbit IgG (H + L) (1:5000, Proteintech, SA00001-2).

### Cell viability assay

Cell viability was assessed using a CCK8 Kit (Biosharp, BS350B). Cells were evenly seeded in 96-well plates, and 100 µL of PBS was added around each well to prevent uneven medium evaporation. After incubating for 12 h, cells were treated as described in the figure legends. Subsequently, 10% CCK-8 reagent was added to each well, and the plates were incubated in a 37 °C, 5% CO_2_ incubator for 1–3 h. Cell activity was determined based on the OD value at a 450 nm wavelength.

### Flow cytometry

Dihydroethidium (DHE) from AppLYGEN (catalog number C1300-2) was utilized to measure ROS levels, while lipid ROS levels were determined using BODIPY™ 581/591 C11 from Thermo Fisher Scientific (catalog number D3861). Necroptosis, on the other hand, was measured using Annexin V-FITC/PI kit from Vazyme (catalog number A211-01). Each group was washed in ice-cold PBS at 2000 rpm for 5 min twice. Then, cells were treated according to instructions in a dark environment for 30–60 min. The cells were washed and suspended in PBS before being loaded onto a flow cytometer and analyzed using FlowJo software.

### Immunofluorescent staining

Cells were washed with PBS at 1000 rpm for 5 min twice, fixed with 4% paraformaldehyde for 2 h, then permeabilized with 0.1% Triton X-100 for 10 min at room temperature. After centrifugation, the supernatant was discarded, and the cells were washed and treated as previously described. 1% bovine serum albumin (BSA) was used for blocking non-specific sites for 1 h and mixed every 10 min at room temperature. After centrifugation at 5000 rpm for 10 min, cells were incubated with primary antibodies FSP1 (1:500, Proteintech, 20886-1-AP) and importin α2 (1:200, Santa Cruz, SC-55538) at 4 °C overnight. Then, cells were extracted and washed three times, followed by centrifugation at 4 ℃, 5000 rpm for 10 min. Next, cells were treated with the corresponding secondary antibodies, including Cy3 (1:250, Proteintech, SA00009-2) and FITC (1:250, Sigma, F0257) for 1 h in a dark room. DAPI (4A BIOTECH, FXP139) or Hochest3344 (Solarbio, B8040) were applied to stain nuclei. Images were acquired with a ZEISS fluorescence microscope and a confocal microscope system.

### Real-time quantitative PCR

Following drug treatments, total RNA was extracted from THP-1 cells using Trizol reagent (Biosharp, BS259A) and subsequently reverse transcribed into cDNA using the RevertAid First Strand cDNA Synthesis Kit (Thermo Fisher Scientific, K1691). Real-time quantitative PCR (RT-qPCR) was conducted using the Universal SYBR Green Fast qPCR Mix (ABclonal, RK21203) and the QuantStudio™ 3 Real-Time PCR system (Thermo Fisher Scientific, A28136). Expression levels were normalized to the internal control GAPDH. The human primer sets used are listed below.

**Table:** 

Primer	Forward	Reverse
GPX4	5′-GAGGCAAGACCGAAGTAAACTAC-3′	5′-CCGAACTGGTTACACGGGAA-3′
ACSL4	5′-GAATGGATGATTGCAGCACAGA-3′	5′-CCTCAGATTCATTTAGCCCATGAAC-3′
SLC7A11	5′-TCTCCAAAGGAGGTTACCTGC-3′	5′-AGACTCCCCTCAGTAAAGTGAC-3′
PTGS2	5′-ATGCTGACTATGGCTACAAAAGC-3′	5′-TCG GGCAATCATCAGGCAC-3′
P53	5′-CAGCACATGACGGAGGTTGT-3′	5′-TACTCCAAATACTCCACACGC-3′
FTH	5′-GAACTACCACCAGGACTC-3′	5′-TTCTTCAAAGCCACATCATC-3′
NRF2	5′-TCTGACTCCGGCATTTCACT-3′	5′-GGCACTGTCTAGCTCTTCCA-3′
FSP1	5′-ATGGTTCGGCTGACCAAGAG-3′	5′-GCCACCACATCATTGGCATC-3′
Importin α2	5′-GCATAAATAGCAGCAATGTGGA-3′	5′-GGGGCTGTTTTTCTCTGGA-3′
TFR1	5′-GAGCGTCGGGATATCGGGT-3′	5′-CAGGATGAAGGGAGGACACG-3′
Caspase-3	5′-TGTTTGTGTGCTTCTGAGCC-3′	5′-CACGCCATGTCATCATCAAC-3′
Bcl2	5′-ATGTGTGTGGAGACCGTCAA-3′	5′-GCCGTACAGTTCCACAAAGG-3′
Bax	5′-ATGTTTTCTGACGGCAACTTC-3′	5′-AGTCCAATGTCCCAGCCCAT-3′
RIPK3	5′-CAGTGTGCAACAGGCAGAAC-3′	5′-TCAGTCCTTCTAAGCCGGGA-3′
RIPK1	5′-CACAAGGACCTGAAGCCTGAA-3′	5′-TGCTTGTTTTGAGCTGTAGCC-3′
Caspase-8	5′-CCTCATCAATCGGCTGGAC-3′	5′-ATGACCCTGTAGGCAGAAACC-3′
MLKL	5′-AGGAGGCTAATGGGGAGATAGA-3′	5′-TGGCTTGCTGTTAGAAACCTG-3′
GAPDH	5′-TGCACCACCAACTGCTTAGC-3′	5′-GGCATGGACTGTGGTCATGAG-3′

### *Electron* microscopy

Cells were washed with PBS and centrifuged at 2000 rpm for 5 min. Then, the sediment was resuspended in 2.5% glutaraldehyde electron microscope fixative (PUMOKE, PMK0243) for 2 h at 4 ℃. Then, cells were treated according to standard procedures, including post-fixing, dehydrating, embedding, cutting, and double staining with 3.3% uranium-citric acid by Wuhan servicebio. Transmission electron microscopy was used to observe subcellular structures and image acquisition. A minimum of five images were obtained for every structure of interest, and the representative images were shown.

### Evaluation of malondialdehyde (MDA)

To evaluate the level of lipid peroxidation products MDA, cells were washed with PBS and centrifuged at 2000 rpm for 5 min thrice. Then, cells were ultrasonically disintegrated once for 59 s after cell lysate treatment. Next, the cells were further fragmented on ice for 30 min. Proteins were obtained by centrifugation at 12000 rpm for 10 min and were detected by a BCA protein assay kit (CWBIO, CW0014S). The MDA concentration in cell lysates was assessed by a cell malondialdehyde (MDA) assay kit (Nanjing Jiancheng Bioengineering Institute, A003-4-1).

### Evaluation of 4-HNE

The 4-HNE concentration in THP-1 cells was detected by a human 4-HNE Elisa kit (Feiya Biotechnology, FY1914-A). The cells were washed with PBS twice before being lysed with a buffer containing 1% PMSF and sonicated to extract the protein. The protein concentration was then measured. Next, both protein and prepared standard samples were added sequentially to the enzyme standard plate following the instructions provided in the kit. The plate was kept in an incubator at 37 ℃ for 30 min, followed by five washes with a detergent. Next, the enzyme label reagent was added to the plate and reacted at 37 ℃ for 30 min. After washing five times, developer A and developer B were added to each well at 37 ℃ for 10 min. Finally, the OD value of each well was detected at 450 nm wavelength within 15 min after adding the stop solution.

### Evaluation of GSH and GSSG

For the evaluation of 4-HNE, THP-1 cell lysates were prepared as previously mentioned. The relative GSSG and GSH level in lysates was assessed by the T-GSH/GSSG assay kit (Nanjing Jiancheng Bioengineering Institute, A061-1-2). Reagent I, standards of GSH and samples, and reagents II and III were sequentially added into the 96-well plate. 5 min later, the OD value of T-GSH was detected at 405 nm wavelength. Standards of GSSG and samples, reagent V and reagent VI, were added in a 1.5 mL centrifuge tube at 37 ℃ for 30 min. Then, GSSG was detected according to the manufacturer’s instructions. The level of GSH was calculated by T-GSH and GSSG.

### Co-immunoprecipitation

Cells were washed with PBS three times and resuspended in 200–300 μL lysis buffer containing 1%PMSF (Beyotime, P0013J). To extract the protein, ultrasound was used for a 20-s burst followed by resting the sample on ice for 30 min. Next, the protein sample was incubated with an Imp-α2 antibody at 4 °C overnight. Subsequently, the sample was incubated with beads that had been washed with PBS three times, incubated at 4 °C for 12 h. The immunocomplexes were eluted using a loading buffer. Finally, the samples were used to detect the interaction between FSP1 and Imp-α2.

### Statistical analysis

GraphPad Prism 8 software was utilized for data visualization, and the outcomes were presented as means ± standard deviation (SD). Statistical significance among all experimental samples was determined as *P* < 0.05 and was computed using either a One-Way ANOVA.

## Data Availability

The datasets used and/or analysed during the current study are available from the corresponding author on reasonable request.
